# Genetic Associations of Circulating Cardiovascular Proteins with Gestational Hypertension and Preeclampsia

**DOI:** 10.1001/jamacardio.2023.4994

**Published:** 2024-01-03

**Authors:** Art Schuermans, Buu Truong, Maddalena Ardissino, Rohan Bhukar, Eric A. W. Slob, Tetsushi Nakao, Jacqueline S. Dron, Aeron M. Small, So Mi Jemma Cho, Zhi Yu, Whitney Hornsby, Tajmara Antoine, Kim Lannery, Darina Postupaka, Kathryn J. Gray, Qi Yan, Adam S. Butterworth, Stephen Burgess, Malissa J. Wood, Nandita S. Scott, Colleen M. Harrington, Amy A. Sarma, Emily S. Lau, Jason D. Roh, James L. Januzzi, Pradeep Natarajan, Michael C. Honigberg

**Affiliations:** 1 Program in Medical and Population Genetics and Cardiovascular Disease Initiative, Broad Institute of Harvard and MIT, Cambridge, MA, USA; 2 Cardiovascular Research Center, Massachusetts General Hospital, Boston, MA, USA; 3 Department of Cardiovascular Sciences, KU Leuven, Leuven, Belgium; 4 BHF Cardiovascular Epidemiology Unit, Department of Public Health and Primary Care, University of Cambridge, Cambridge, UK; 5 National Heart and Lung Institute, Imperial College London, London, United Kingdom; 6 MRC Biostatistics Unit, University of Cambridge, Cambridge, UK; 7 Department of Applied Economics, Erasmus School of Economics, Erasmus University Rotterdam, Rotterdam, The Netherlands; 8 Erasmus University Rotterdam Institute for Behavior and Biology, Erasmus University Rotterdam, Rotterdam, The Netherlands; 9 Department of Medical Oncology, Dana-Farber Cancer Institute, Boston, MA, USA; 10 Division of Cardiovascular Medicine, Department of Medicine, Brigham and Women’s Hospital, Boston, MA, USA; 11 Department of Medicine, Harvard Medical School, Boston, MA, USA; 12 Integrative Research Center for Cerebrovascular and Cardiovascular Diseases, Yonsei University College of Medicine, Seoul, Republic of Korea; 13 Division of Maternal-Fetal Medicine, Brigham and Women's Hospital, Boston, MA, USA; 14 Department of Obstetrics and Gynecology, Columbia University, New York, NY, USA; 15 BHF Cardiovascular Epidemiology Unit, Department of Public Health and Primary Care, University of Cambridge, Cambridge, UK; 16 BHF Centre of Research Excellence, University of Cambridge, Cambridge, UK; 17 National Institute for Health Research Cambridge Biomedical Research Centre, University of Cambridge and Cambridge University Hospitals, Cambridge, UK; 18 Health Data Research UK Cambridge, Wellcome Genome Campus and University of Cambridge, Cambridge, UK; 19 National Institute for Health Research Blood and Transplant Research Unit in Donor Health and Genomics, University of Cambridge, Cambridge, UK; 20 Cardiology Division, Massachusetts General Hospital, Boston, MA, USA; 21 Lee Health, Fort Myers, FL, USA; 22 Baim Institute for Clinical Research, Boston, MA, USA

## Abstract

**Importance:**

Hypertensive disorders of pregnancy (HDPs; including gestational hypertension and preeclampsia) are important contributors to maternal morbidity and mortality worldwide. In addition, women with HDPs face an elevated long-term risk of cardiovascular disease.

**Objective:**

To identify proteins in the circulation associated with HDPs.

**Design:**

Mendelian randomization (MR) tested the associations of genetic instruments for cardiovascular disease-related proteins with gestational hypertension and preeclampsia. In downstream analyses, we conducted a systematic review of observational data to evaluate the identified proteins' dynamics across gestation in hypertensive vs. normotensive pregnancies and performed phenome-wide MR analyses to identify potential non-HDP-related effects associated with the prioritized proteins.

**Setting:**

Two-sample MR analysis.

**Participants:**

Genetic association data for cardiovascular disease-related proteins were obtained from the SCALLOP consortium, including 21,758 European-ancestry participants. Genetic association data for the HDPs were obtained from recent genome-wide association study meta-analyses, including 393,238 females (8,636 cases and 384,602 controls) for gestational hypertension and 606,903 (16,032 cases and 590,871 controls) for preeclampsia.

**Exposures:**

Genetic instruments for 90 candidate proteins implicated in cardiovascular diseases, constructed using *cis*-protein quantitative trait loci (*cis*-pQTLs).

**Main Outcomes and Measures:**

Gestational hypertension and preeclampsia.

**Results:**

Seventy-five of 90 proteins had at least one valid *cis*-pQTL. Of those, ten proteins were significantly associated with HDPs. Four were robust to sensitivity analyses for gestational hypertension (CD40, ECP, Gal-3, NT-proBNP) and two were robust for preeclampsia (CSTB, HSP27). Consistent with our MR findings, observational data revealed that lower NT-proBNP and higher HSP27 levels early in pregnancy were associated with increased risk of HDPs, as were higher levels of ECP. Phenome-wide MR analyses suggested on-target side effects, both beneficial and adverse, associated with interventions lowering HDP risk through the identified proteins.

**Conclusions and Relevance:**

Our findings identify genetic associations of four cardiovascular disease-related proteins with gestational hypertension and two associated with preeclampsia. Future studies are required to test the efficacy of targeting the corresponding pathways to reduce HDP risk.

## Introduction

The hypertensive disorders of pregnancy (HDPs) are a leading cause of maternal and neonatal morbidity and mortality, affecting up to 15% of child-bearing females and accounting for 14% of maternal deaths worldwide.^1,2^ Gestational hypertension (new-onset hypertension after 20 weeks of gestation) and preeclampsia (gestational hypertension with proteinuria or other maternal end-organ dysfunction) account for approximately 90% of hypertensive pregnancies.^2,3^ In addition to the immediate maternal and neonatal complications of HDPs, affected individuals also face an increased long-term risk of cardiovascular events and premature mortality.^2,4,5^ Given the significant impact of HDPs on maternal and neonatal health, there is currently an unmet need for new therapeutics to prevent and treat these conditions.

The cardiovascular system plays a central role in the onset of HDPs.^6,7^ For example, in preeclampsia, defective placental implantation and abnormal remodeling of the uterine spiral arteries lead to impaired placental perfusion later in gestation, which—in turn—leads to angiogenic factor imbalance, endothelial dysregulation, and systemic vasoconstriction. Consistent with this framework, genome-wide association studies (GWAS) suggest that most genetic loci associated with gestational hypertension and/or preeclampsia are related to cardiovascular processes.^7,8^ However, it remains unclear whether cardiovascular disease-related pathways could represent potential drug targets for HDPs.

While current management strategies for HDPs include blood pressure control, seizure prevention, and timed delivery,^6^ none of these interventions targets underlying molecular pathways. This lack of disease-specific pharmacotherapeutic options can be partially ascribed to an incomplete understanding of the molecular mechanisms driving HDPs and challenges associated with drug development for obstetric conditions.^9^ For instance, while aspirin can be used to prevent preterm preeclampsia, mechanisms by which aspirin exerts its prophylactic effects remain unclear.^10^ In addition, traditional methods to identify drug targets such as animal models have often been unsuccessful in capturing the complex pathophysiology underlying HDPs and, consequently, have not translated to effective interventions in clinical trials.^9^

Recent studies have identified genetic variants associated with plasma protein levels (protein quantitative trait loci [pQTLs]),^11^ facilitating the identification of drug targets for human diseases using Mendelian randomization (MR).^12–15^ Given the limitations of traditional methods for identifying HDP drug targets, genetic approaches may help prioritize new therapeutic targets for these conditions. Here, we leveraged MR to identify therapeutic targets for HDPs. We constructed genetic instruments for candidate cardiovascular disease-related plasma proteins and estimated their effects on gestational hypertension and preeclampsia. We evaluated observational associations between prioritized proteins and HDPs and conducted phenome-wide MR analyses to explore potential side effects associated with therapeutically targeting these proteins. Finally, we evaluated the potential druggability of the identified proteins as therapeutic targets for gestational hypertension and preeclampsia.

## Methods

A detailed description of the methods can be found in the eMethods (as well as eFigures 2-3 and eTables 1-6).

### Study Design

The study design is summarized in eFigure 1. We used pQTLs as the exposures throughout all MR analyses. Because the use of *cis*-pQTLs (pQTLs near the protein-encoding gene) facilitates adherence to the core assumptions of MR,^15,16^ all genetic instruments for circulating protein levels were constructed using *cis*-pQTLs (referred to as *cis*-MR). Additional information on the assumptions of MR and the impact of *cis*-pQTLs on those can be found in the eMethods.

### Genome-Wide Association Studies

Genetic association data for circulating protein levels were obtained from a meta-analysis including 21,758 European-ancestry individuals enrolled in 13 cohorts from the SCALLOP consortium.^11^ Approximately 63.1% of participants were recruited in population-based studies, 15.8% in a cohort of participants with metabolic syndrome, 13.8% in a randomized controlled trial of coronary artery disease, 4.1% in a population-based study with oversampling of participants with diabetes, and 3.2% in a case-control study of bipolar disorder (eTable 1).^11^ Biobanks and cohorts contributing data to the HDP GWAS meta-analyses began enrolling participants between 1989-2017; apart from a subset of cohorts contributing to the *InterPregGen* consortium, all other biobanks/cohorts began enrollment after 1999.

Association data for HDPs were obtained from GWAS meta-analyses by Honigberg *et al*.^8^ for gestational hypertension (8,636 cases, 384,602 controls) and preeclampsia/eclampsia (16,032 cases, 590,871 controls). Participants were predominantly recruited from population-based or health system-linked cohort studies, with HDP cases primarily identified using qualifying *International Classification of Diseases* codes or phecodes; controls were primarily those with only normotensive pregnancies or all females without codes for hypertension in pregnancy (eTable 3).^8,17^

### Cis-Mendelian Randomization Analyses

Genetic instruments for plasma proteins were constructed using region-wide significant, largely uncorrelated *cis*-pQTLs (±200 kilobases, *P*<1×10^-4^, *R*^*2*^<0.4 in primary analyses).^15,16^ Primary analyses used the inverse-variance-weighted (IVW) method with fixed effects for instruments with 2-3 variants and multiplicative random effects for instruments with >3 variants. When the instrument included a single variant, we used the Wald ratio method. In addition, to avoid spurious associations due to residual correlation between variants, we adjusted for between-variant correlation structure in all primary IVW models as described previously.^18,19^

Multiple sensitivity analyses were conducted to probe robustness of our findings using different instrument selection parameters and MR methods.^12^ First, because *cis*-MR analyses often rely on variants that are moderately correlated with each other,^20^ we performed MR analyses using different correlation thresholds (*R*^2^<0.001/*R*^2^<0.01/*R*^2^<0.1/*R*^2^<0.2/*R*^2^<0.4/*R*^2^<0.6/*R*^2^<0.8). Second, additional sensitivity analyses used stricter *P*-value thresholds to construct genetic instruments (*P*<1×10^-4^/*P*<1×10^-6^/*P*<5×10^-8^). Third, we conducted analyses using MR models with principal components explaining 99% of the genetic variance.^18,20^ Fourth, we calculated effect estimates using MR-Egger (adjusted for residual correlation between variants), which accounts for horizontal pleiotropy. Finally, we tested for reverse causation by performing Steiger filtering, which removes variants explaining more variance in the outcome than the exposure, and testing the genetic associations of HDPs (exposure) with the indicated proteins (outcome).

Two-sided, false discovery rate (FDR)-adjusted *P*<0.05 was used to define statistical significance for the primary analyses. Associations of proteins with HDPs were considered robust if: (1) the primary analysis was statistically significant; (2) all sensitivity analyses were directionally consistent; and (3) there was no evidence of reverse causation (unadjusted *P*>0.05).

### Downstream Analyses

Downstream analyses further explored the proteins that survived sensitivity analyses. We (1) performed replication analyses using pQTL data from the UK Biobank Pharma Proteomics Project (UKB-PPP); (2) carried out colocalization analyses to test for shared causal variants between the prioritized proteins' *cis* loci and HDPs (3) conducted a systematic review of observational data from to gain insights into the identified proteins' dynamics across gestation in hypertensive vs. normotensive pregnancies; (4) performed phenome-wide MR analyses to identify potential non-HDP-related effects ("on-target side effects"); and (5) evaluated the druggability profiles of all identified target proteins (eMethods).

## Results

### Genetic Associations of Cardiovascular Proteins with Gestational Hypertension and Preeclampsia

Of the 90 candidate proteins, 85 were encoded by autosomal genes and had genetic association data available for the *cis*-regions of interest (eTable 2). Using the instrument selection parameters for our primary analyses (*P*<1×10^-4^, *R*^2^<0.4), genetic instruments were constructed for 75 proteins. The median number of variants included in the genetic instruments was 20 (interquartile range, 7-40). Genetic variants used for each protein-specific genetic instrument are listed in eTable 7. All *F*-statistics were estimated to be >15, suggesting low risk of weak instrument bias.

**Figure 1:**
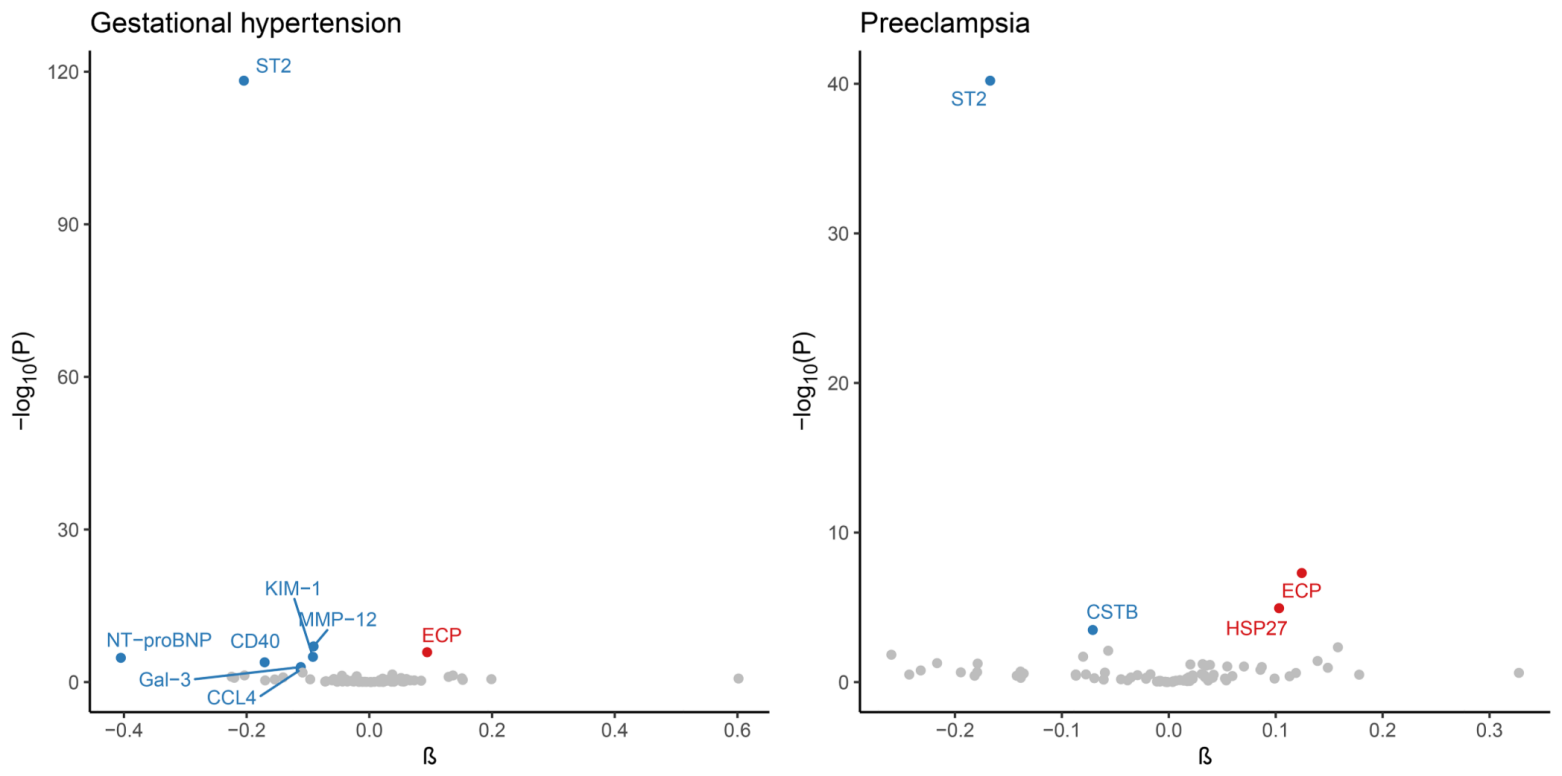
Associations of Genetically Predicted Protein Levels with HDPs in Primary Analyses. *Cis*-MR analyses were performed using *cis*-variants at *P*<1×10^-4^ clumped at *R*^2^<0.4. Associations are expressed per SD increase in genetically predicted protein levels. Biomarkers reaching statistical significance (FDR-adjusted *P*<0.05) are displayed in blue (if *β*<0) or red (if *β*>0).

Primary analyses identified 10 proteins associated with gestational hypertension and/or preeclampsia at FDR-adjusted *P*<0.05. Among those, 8 were associated with gestational hypertension: CCL4 (C-C motif chemokine 4), CD40 (cluster of differentiation 40), ECP (eosinophil cationic protein), Gal-3 (galectin-3), KIM-1 (kidney injury molecule 1), MMP-12 (matrix metalloproteinase-12), NT-proBNP (N-terminal pro-B-type natriuretic peptide), and ST2. Four proteins were associated with preeclampsia: CSTB (cystatin B), ECP, HSP27 (heat shock protein 27), and ST2. For ECP and HSP27, higher genetically predicted levels increased HDP risk, suggesting that higher levels of these proteins can lead to HDPs. The remaining proteins (including NT-proBNP) were negatively associated with HDPs, suggesting that higher levels are protective against HDPs (Figure 1, eTables 8-9).

**Figure 2:**
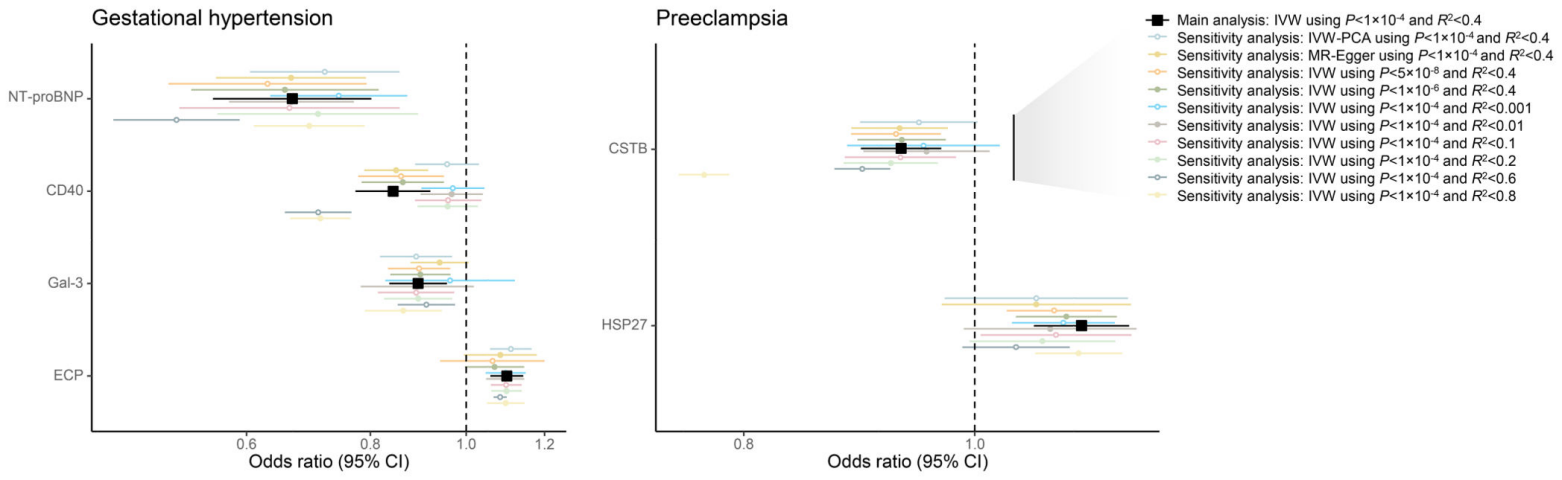
Genetic Associations of Protein Levels with HDPs Robust to Sensitivity Analyses. Forest plots show associations that were significant in primary analyses (dark red, squares) with directionally consistent sensitivity analyses (pink, circles). Associations are expressed per SD increase in genetically predicted protein levels. Main analyses included *cis*-pQTLs with *P*<1×10^-4^ at *R*^2^<0.4 and used IVW adjusting for between-variant correlation. From top to bottom, sensitivity analyses used IVW with principal component analysis (99% of variance; IVW-PCA); MR-Egger; IVW adjusting for between-variant correlation using different linkage disequilibrium *R*^2^ thresholds (0.001, 0.01, 0.1, 0.2, 0.6, and 0.8); and IVW adjusting for between-variant correlation using different *P*-value thresholds (1×10^-6^ and 5×10^-8^).

We performed multiple sensitivity analyses using different selection parameters and MR methods to probe the robustness of our findings (eTable 10). Robust associations (i.e., directional consistency across all sensitivity analyses) were observed for 4 of 8 proteins associated with gestational hypertension (CD40, ECP, Gal-3, and NT-proBNP) and 2 of 4 proteins associated with preeclampsia (CSTB and HSP27; Figure 2). All robustly associated proteins, except CD40 for gestational hypertension and CSTB for preeclampsia, had directionally consistent associations with the other HDP subtype (eTables 8-9). ECP, which was robust to all sensitivity analyses for gestational hypertension, was also robust to all but one sensitivity analysis for preeclampsia. Steiger filtering did not identify any *cis*-variants explaining more variance in the outcome (HDPs) than the exposure (protein levels) for most biomarkers; HSP27—the only protein with "reverse causal" variants—was still strongly associated with preeclampsia after excluding a single variant identified using Steiger filtering (*β*, 0.12 [95%CI, 0.08-0.17]; *P*=1.1×10^-8^). Similarly, MR analyses testing the opposite direction of effects all yielded unadjusted *P*-values >0.05, further suggesting no bias from reverse causation (eTable 11).

All 6 robust associations replicated with *P*<1×10^-4^ using pQTLs derived from the UK Biobank (eTable 12).^21^ Colocalization was inconclusive for most proteins under study (eTable 13). There was strong evidence of a shared causal variant between Gal-3 and gestational hypertension in the UKB-PPP (posterior probability for H_4_ >0.80). Colocalization evidence for NT-proBNP was mixed, with strong evidence of colocalization when examining variants within the *NPPB* gene but suggestion of distinct causal variants when broadening to a window of ±200 kilobases.

### Observational Associations of Target Proteins with Gestational Hypertension and Preeclampsia

Observational studies suggest that the magnitude and direction of associations between HDPs and circulating proteins can change across gestation.^22^ To gain insights into the identified proteins' dynamics during hypertensive vs. normotensive pregnancies, we performed a systematic review of studies reporting observational associations of the prioritized proteins with HDPs. Forty-three studies met our inclusion criteria (eFigure 3), encompassing 9,749 pregnant individuals with protein measurements who enrolled from 1998 to 2020 in their respective studies. Of those, 3,122 (32.0%) experienced an HDP, including 939 (9.6%) with gestational hypertension, 2,167 (22.2%) with preeclampsia, and 16 (0.2%) without information on HDP subtype. The most frequently tested biomarker was NT-proBNP (*n*=8,940; 30 studies), followed by Gal-3 (*n*=921; 11 studies), HSP27 (*n*=363; 5 studies), and CD40 (*n*=81; 2 studies). CSTB and ECP were both evaluated by a single study including 66 participants. Detailed information on each study's design and participants can be found in eTables 14-15.

**Figure 3:**
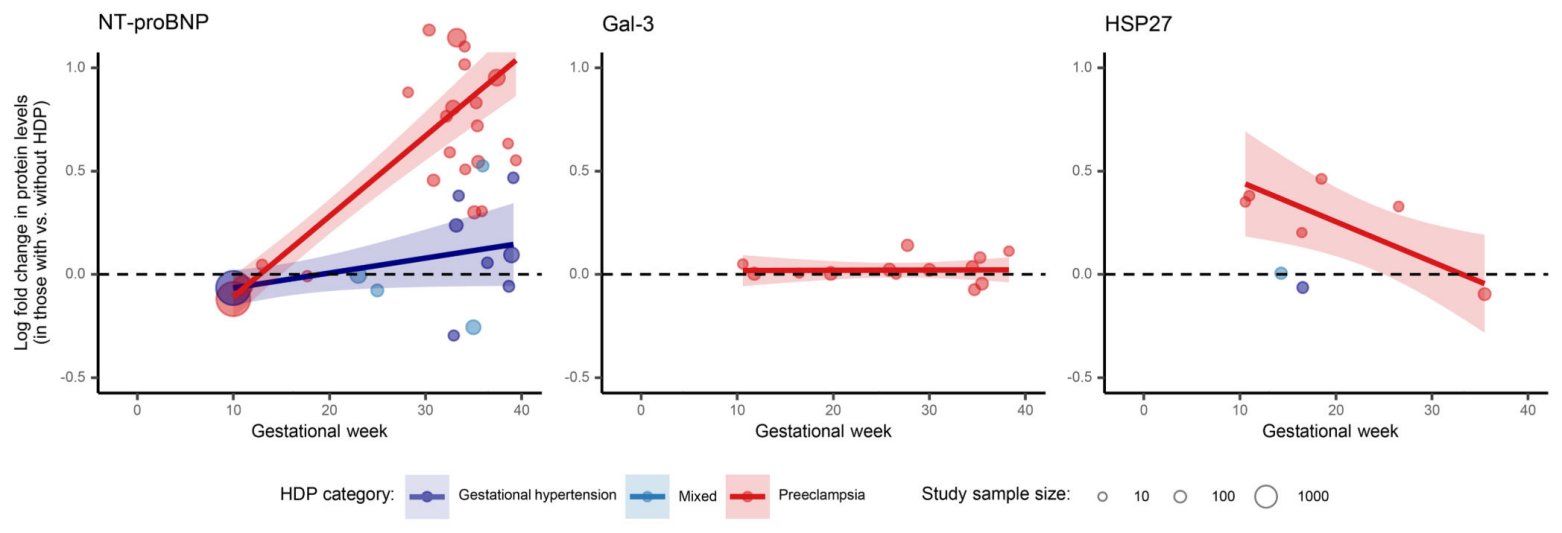
Observational Associations Between HDPs and NT-proBNP, Gal-3, and HSP27 Across Gestation. Scatter plots illustrate the relationship between protein levels and gestational age at blood sampling. Protein levels were compared by log_10_-transforming the ratio of mean protein concentration in the HDP vs. non-HDP group. Lines depict linear regression estimates (and corresponding 95% confidence bands) for HDP subgroups (“preeclampsia” or “gestational hypertension”) weighted by each study's sample size, generated using *ggplot2* in R. Studies with data labeled as “mixed” did not distinguish between preeclampsia and gestational hypertension. Each 1-week increase in gestational age was associated with a 0.039-point increase (95%CI, 0.032 to 0.046; *P*=4.4×10^-10^) in NT-proBNP abundance (log fold change in women with vs. without HDP) for preeclampsia; a 0.007-point increase (95%CI, -0.000 to 0.015; *P*=0.06) in NT-proBNP abundance for gestational hypertension; a 0.000-point increase (95%CI, -0.003 to 0.004; *P*=0.94) in Gal-3 abundance for preeclampsia; and a 0.019-point decrease (*β*, -0.019 [95%CI, -0.035 to -0.005]; *P*=0.03) in HSP27 abundance for preeclampsia.

Information on observational protein levels in pregnant individuals with vs. without HDPs is provided in eTable 16. In contrast with the established associations of higher NT-proBNP levels with cardiac dysfunction and heart failure,^23^ lower first-trimester NT-proBNP was associated with subsequent development of HDPs. The direction of this association reversed during the second and third trimesters of pregnancy, with higher NT-proBNP levels in those with vs. without HDPs (Figure 3A). We did not observe a similar temporal trend for Gal-3 in individuals with preeclampsia (Figure 3B). There were no data available on Gal-3 in individuals with gestational hypertension. For HSP27, higher levels early in pregnancy were associated with the subsequent development of preeclampsia (Figure 3C). Temporal trends across gestation were observed for NT-proBNP and HSP27 in both linear and nonlinear models (eFigure 4). CD40 and ECP, although only measured in <100 participants each, were higher among participants with preeclampsia vs. no HDPs (eTable 16). Overall, observational data were available and consistent with MR analyses for 3 out of 6 prioritized biomarkers (NT-proBNP, HSP27, and ECP), including both biomarkers (NT-proBNP and HSP27) with available first trimester data.

### Phenome-Wide Mendelian Randomization Analyses of Therapeutic Targets

Next, we performed phenome-wide MR analyses to investigate potential non-HDP-related effects (i.e., "on-target side effects") associated with therapeutic targeting of the identified proteins. Utilizing a lenient significance threshold of *P*<0.0083 (i.e., *P*<0.05/6 [for 6 tested proteins]), which may increase the sensitivity to detect potential side effects but may also lead to more false-positive findings, we identified 37 unique protein-disease associations (eTables 17-18). Among these, 17 (45.9%) were beneficial, indicating that therapeutic targeting of these proteins to reduce HDP risk was associated with a lower risk of the corresponding diseases. Musculoskeletal disorders constituted the most frequently implicated phecode-based disease category (*n*=8/37 [21.6%]).

Table 1 summarizes protein-specific findings from our phenome-wide MR analyses. Gal-3 had the highest number of potential on-target side effects (*n*=13), the majority of which were adverse (*n*=9/13 [69.2%]). Each SD increase in genetically predicted protein levels was associated with 1.20-fold odds of having upper respiratory tract infections (*β*, 0.18 [95%CI, 0.09-0.28]; *P*=2.2×10^-4^), consistent with clinical trials testing Gal-3 inhibitors for the treatment of respiratory tract infections.^24^ NT-proBNP had the fewest disease associations, with only a single beneficial association identified: each SD increase in genetically predicted levels was associated with 0.58-fold odds of having edema symptoms (*β*, -0.55 [95%CI, -0.84 to -0.26] per SD increase in genetically predicted protein levels; *P*=2.4×10^-4^).

### Druggability of Potential Therapeutic Targets

To determine whether the identified proteins could serve as therapeutic targets for gestational hypertension and/or preeclampsia, we extracted their druggability profiles from a recently published list of druggable genes.^25^ All prioritized proteins except CSTB were considered druggable (Table 2, eTable 19).

## Discussion

We used MR to test the genetic associations of various candidate cardiovascular disease-related proteins with gestational hypertension and preeclampsia. Primary analyses identified 10 proteins reflecting pathways with potential roles in the development of HDPs, 6 of which were robust to sensitivity analyses for gestational hypertension (CD40, ECP, Gal-3, NT-proBNP) or preeclampsia (CSTB, HSP27). Consistent with these findings, observational data revealed that pregnant individuals with lower NT-proBNP and higher HSP27 levels during early gestation were at a higher risk of experiencing HDPs, as were those with higher levels of ECP. Phenome-wide MR analyses suggested potential on-target side effects, both beneficial and adverse, associated with interventions to lower HDP risk through the identified proteins. Collectively, these findings provide insights into biological mechanisms and identify potential therapeutic targets for HDPs.

First, our findings identify natriuretic peptide signaling as a potential therapeutic target for HDPs. NT-proBNP and BNP, members of the natriuretic peptide family, are derived from a common prohormone (proBNP) encoded by the *NPPB* gene.^26^ ProBNP is primarily synthesized and secreted by cardiac myocytes in response to increased myocardial wall tension, after which it is cleaved in equimolar quantities into inert NT-proBNP and bioactive BNP, which enhances natriuresis and reduces vascular tone. NT-proBNP levels change during uncomplicated pregnancies: they increase during the first trimester and decline thereafter,^27^ likely reflecting physiological adaptations to volume expansion early in gestation. Recent data from the nuMoM2b study, a large prospective U.S. cohort study of pregnant individuals, revealed that lower first trimester NT-proBNP levels were associated with increased risks of gestational hypertension, preeclampsia, and hypertension after delivery.^28^ Our *cis*-MR analyses affirm and extend these findings by demonstrating that lower genetically predicted NT-proBNP levels are associated with increased risk of developing gestational hypertension. Furthermore, genetic studies implicate lower expression of *NPPA* (which encodes atrial natriuretic peptide [ANP] and has strong shared genetic regulation mechanisms with *NPPB*^29^) in the development of HDPs,^8^ with ANP-deficient mice demonstrating impaired trophoblast invasion and uterine spiral artery remodeling.^30,31^ These findings collectively suggest that the HDPs may represent a syndrome of deficient natriuretic peptide signaling, potentially implicating a paradigm of cardiac-placental crosstalk underlying the core pathobiology of HDPs. Importantly, designer natriuretic peptides mimicking the effects of BNP or ANP are currently under development for cardiovascular diseases such as hypertension and heart failure.^26^ Future studies are required to test the effectiveness of direct modulation (e.g., designer natriuretic peptides), indirect modulation, or tailored management (e.g., conservative fluid management in high-risk patients) of these pathways to prevent the onset and/or long-term cardiovascular consequences of HDPs.

Second, our findings provide novel insights into inflammatory mechanisms underlying HDPs. Specifically, we observed that higher genetically predicted ECP levels increased the risk of gestational hypertension. ECP is a cytotoxic protein involved in immune regulation and serves as an established biomarker for eosinophil activation.^32^ Previous observational and MR studies have indicated that ECP plays a role in the onset and progression of asthma,^32^ a known risk factor for HDPs.^33^ As recent research implicates a role for ECP in atherogenesis and vascular calcification,^34^ it is possible that ECP contributes to accelerated atherosclerosis in individuals with prior HDPs.^2,5^ Furthermore, we also identified HSP27—an intracellular protein involved in stress response and cell survival—as a potentially causal biomarker for preeclampsia. When released extracellularly, HSP27 promotes inflammation through increased expression of IL-1β and TNF-α.^35^ Experiments in mice indicate that HSP27 is upregulated from conception to delivery in response to physiologic stress associated with pregnancy.^36^ It has been proposed that homeostasis of extracellular heat shock proteins is important for immune tolerance during pregnancy, with increased heat shock protein levels predisposing to an immunogenic rather than tolerant phenotype toward the fetus.^37^ Consistent with this framework, human genetic data suggest that heat shock proteins are important contributors to spontaneous preterm delivery.^38^ These data, together with the genetic and observational findings from the present study, suggest a role for HSP27 in pregnancy-associated inflammation.

Third, this study corroborates the notion that the relevance of circulating proteins with HDPs can change throughout gestation. Pregnancy is a dynamic process, reflected by longitudinal changes in the plasma concentrations of certain proteins.^22,27^ Previous research has shown that associations of placental proteins with HDPs change throughout pregnancy.^22^ The present analysis extends these findings by demonstrating that the direction of observational biomarker associations with HDPs may reverse between early and late pregnancy. In addition to longitudinal changes throughout gestation, emerging evidence suggests a complex interplay between fetal- and maternally-encoded proteins in human pregnancy.^39^ Further research is necessary to elucidate the relative contributions of other fetal- and maternal-encoded proteins to the development of HDPs, underscoring the need for additional efforts (e.g., regulatory incentives) to include pregnant individuals at various stages of gestation in clinical research.

### Study Limitations

While our study benefits from large genetic datasets and a robust *cis*-MR framework, findings must be interpreted in the context of limitations. First, we only examined 90 proteins within the Olink CVD-I panel; this targeted approach has advantages but only examines candidate proteins. Second, our analysis only included genetic instruments identified in European-ancestry cohorts, limiting generalizability to other ancestries. Similarly, there was limited racial/ethnic diversity among the studies included in our systematic review. While data from the nuMoM2b study suggest that the associations of low NT-proBNP levels early in pregnancy with the subsequent development of HDPs and hypertension after delivery are similar across self-reported races/ethnicities,^28^ further studies are warranted to evaluate potential differences across races/ethnicities. Third, although MR can be used to infer causality in given exposure-outcome relationships, any causal inference relies on the justification of the underlying MR assumptions. The present study used a robust *cis*-MR framework facilitating adherence to these assumptions,^12–14^ probed the robustness of the study findings through multiple sensitivity and replication analyses, and found no substantial evidence of pleiotropic associations. This supports the notion that the identified proteins are causally implicated in HDPs. Nevertheless, candidate therapeutic targets remain to be validated in intervention trials, and additional efforts are needed to overcome barriers to the inclusion of pregnant individuals in scientific trials and further scientific progress on reducing pregnancy complications.^40^ Fourth, genetic association data for HDPs were predominantly based on diagnostic code-based definitions, the use of which may differ across studies and change over time. Finally, we constructed genetic instruments using pQTLs derived from non-pregnant individuals.^11^ While we speculate that our analysis (using pQTLs from the general population) may more closely represent first-trimester biology, the genetic regulation of plasma proteins during pregnancy has not been studied at scale. Recent data suggest that between-sex differences in pQTLs are limited^41,42^ with few sex-specific effects on protein-disease associations,^43^ but whether sex-stratified pQTLs may yield additional insights warrants future investigation. However, our MR findings are consistent with observational associations between HDPs and first-trimester protein levels for NT-proBNP and HSP27, suggesting that pQTLs derived from non-pregnant individuals can recapitulate associations between proteins and outcomes in pregnancy.

## Conclusions

Although there are currently no pharmacotherapeutic options available that specifically target the underlying causal pathways leading to HDPs, disease-specific therapeutics could potentially benefit many high-risk pregnant individuals. Here, we used MR to infer associations of various candidate proteins with gestational hypertension and preeclampsia. Our analysis revealed druggable proteins involved in cardiovascular and inflammatory processes. Future studies should evaluate the efficacy of targeting these pathways in animal models and human trials.

## Supplementary Material

Supplementary material

Supplementary tables

## Figures and Tables

**Table 1:** Summary of Phenome-Wide Mendelian Randomization (MR) Analyses Evaluating Potential On-Target Side Effects Associated with Therapeutic Interventions on the Identified Proteins.

Circulating protein	Number of potential beneficial or adverse side effects, *N*	Beneficial effects, *n* (%)	Strongest associations (beneficial or adverse)
CD40 (cluster of differentiation 40)	5	4 (80.0%)	Hemoptysis (beneficial); non-Hodgkin lymphoma (beneficial); back pain (beneficial)
CSTB (cystatin B)	5	3 (60.0%)	Melanoma (adverse); viral hepatitis (beneficial); infections of skin and subcutaneous tissue (beneficial)
ECP (eosinophil cationic protein)	9	5 (55.6%)	Glaucoma (adverse); intestinal obstruction (beneficial); inguinal hernia (beneficial)
Gal-3 (galectin-3)	13	3 (23.1%)	Upper respiratory tract disease (adverse); ganglion/cyst of synovium, tendon, or bursa (adverse); osteoarthrosis (adverse)
HSP-27 (heat shock protein 27)	4	1 (25%)	Acute/chronic tonsillitis (adverse); acquired toe deformities (beneficial); age-related cataract (adverse)
NT-proBNP (N-terminal pro-brain natriuretic peptide)	1	1 (100%)	Edema (beneficial)

Protein-disease associations were considered significant if the IVW method (correcting for between-variant correlation structure) yielded a *P*<0.0083 (*P*<0.05/6). A protein-disease association was considered "beneficial" if genetically predicted alterations in protein levels, consistent with reduced HDP risk, were associated with a lower risk of the corresponding phecode-based disease phenotype.

**Table 2:** Druggability of the Identified Proteins Representing Therapeutic Targets for Gestational Hypertension and/or Preeclampsia.

Gene	Corresponding circulating protein	Druggability	Clinical development status	Molecule type	Compound names
*CD40*	CD40 (cluster of differentiation 40)	Listed as druggable	Target of clinical-phase drug candidates (phase I and II)	Biotherapeutics (antibodies, recombinant ligands)	CDX-1140, cifurtilimab, giloralimab, mitazalimab, recombinant CD40 ligand, selicrelumab, sotigalimab
*CSTB*	CSTB (cystatin B)	Not currently listed as druggable	N/A	N/A	N/A
*RNASE3*	ECP (eosinophil cationic protein)	Listed as druggable	Not a current target of clinically approved compounds or clinical-phase drug candidates	Biotherapeutics	N/A
*LGALS3*	Gal-3 (galectin-3)	Listed as druggable	Not a current target of clinically approved compounds or clinical-phase drug candidates	Biotherapeutics	N/A
*HSPB1*	HSP-27 (heat shock protein 27)	Listed as druggable	Target of clinical-phase drug candidates (phase I and II)	Small molecules	Apatorsen
*NPPB*	NT-proBNP (N-terminal pro-brain natriuretic peptide)	Listed as druggable	Not a current target of clinically approved compounds or clinical-phase drug candidates	Biotherapeutics	N/A
*NPR1* ^a^	GC-A (particulate guanylyl cyclase receptor A)	Listed as druggable	Target of clinically approved compounds and clinical-phase drug candidates (phase I and II)	Biotherapeutics and small molecules	ANX-042, cenderitide, CRRL408, MANP, nesiritide, PL-3994

a Designer natriuretic peptides targeting GC-A (encoded by *NPR1*) have mechanisms that align with higher NT-proBNP levels.

## Data Availability

Genetic association data for circulating proteins were obtained from the SCALLOP consortium (https://doi.org/10.5281/zenodo.2615265).^11^ Genetic association data for HDPs were obtained using European-ancestry summary statistics from Honigberg *et al*.^8^ MR analyses were performed using the *TwoSampleMR* and *MendelianRandomization* packages in R.^44,45^

## Key Points

### Question

Can Mendelian randomization identify associations between circulating cardiovascular disease-related proteins and hypertensive disorders of pregnancy (HDPs)?

### Findings

Using genetic variants associated with circulating proteins as instrumental variables, we identified 6 biomarkers with robust genetic associations with gestational hypertension and/or preeclampsia representing different pathways (e.g., natriuretic peptide signaling, inflammation). Observational data were consistent with Mendelian randomization results for several proteins, with dynamic associations of these proteins with HDPs throughout gestation.

### Meaning

This study highlights novel biological mechanisms and identifies potential therapeutic targets for HDPs.
