# Supplementary material for: Genetic Associations of Circulating Cardiovascular Proteins with Gestational Hypertension and Preeclampsia
Source: JAMA Cardiol. Author manuscript; Available in PMC 2024 Mar 13. (PMC10765315; doi:10.1001/jamacardio.2023.4994)
Supplement: Supplementary material [file EMS194256-supplement-Supplementary_material.docx]

**SUPPLEMENTAL MATERIAL**

*Genetic Associations of Circulating Cardiovascular Proteins with Gestational Hypertension and Preeclampsia*

Art Schuermans, Buu Truong, Maddalena Ardissino, Rohan Bhukar, Eric A. W. Slob,

Tetsushi Nakao, Jacqueline S. Dron, Aeron M. Small, So Mi Jemma Cho, Zhi Yu,

Whitney Hornsby, Tajmara Antoine, Kim Lannery, Darina Postupaka, Kathryn J. Gray,

Qi Yan, Adam S. Butterworth, Stephen Burgess, Malissa J. Wood, Nandita S. Scott,

Colleen M. Harrington, Amy A. Sarma, Emily S. Lau, Jason D. Roh, James L. Januzzi Jr., Pradeep Natarajan, Michael C. Honigberg

**TABLE OF CONTENTS**

[eMethods 5](#_Toc146972306)

[Assumptions of MR and the use of *cis*-pQTLs 5](#_Toc146972307)

[Measurement and GWAS of plasma protein levels 5](#_Toc146972308)

[GWAS of gestational hypertension and preeclampsia 6](#_Toc146972309)

[Construction of genetic instruments 6](#_Toc146972310)

[*Cis-*Mendelian randomization analyses 7](#_Toc146972311)

[Sensitivity analyses 7](#_Toc146972312)

[Replication analyses in the UK Biobank Pharma Proteomics Project 8](#_Toc146972313)

[Colocalization analyses 9](#_Toc146972314)

[Systematic review of observational data 9](#_Toc146972315)

[Phenome-wide MR analyses 10](#_Toc146972316)

[Assessment of druggability profiles 10](#_Toc146972317)

[Statistical software and packages 11](#_Toc146972318)

[eFigures 12](#_Toc146972319)

[eFigure 1. Study design. 12](#_Toc146972320)

[eFigure 2. Schematic illustration of the *cis*-Mendelian randomization (*cis*-MR) framework. 13](#_Toc146972321)

[eFigure 3. Flow diagram of the literature search performed to identify studies reporting observational associations of CD40, CSTB, ECP, Gal-3, HSP-27, or NT-proBNP with hypertensive disorders of pregnancy (HDPs). 14](#_Toc146972322)

[eFigure 4. Observational associations between HDPs and NT-proBNP, Gal-3, and HSP27 across gestation, modeled using loess-smoothed averages. 15](#_Toc146972323)

[eTables (legends) 16](#_Toc146972324)

[eTable 1. Description of cohorts included in the GWAS meta-analysis of circulating protein levels from the SCALLOP consortium. 16](#_Toc146972325)

[eTable 2. Description of Olink CVD-I proteins included in the present study. 16](#_Toc146972326)

[eTable 3. Description of cohorts included in the GWAS meta-analysis of gestational hypertension and preeclampsia/eclampsia. 16](#_Toc146972327)

[eTable 4. Characteristics of UK Biobank Pharma Proteomics Project (UKB-PPP) participants who provided genetic data for replication analyses. 16](#_Toc146972328)

[eTable 5. Systematic search strategy used to identify studies reporting observational associations of CD40, CSTB, ECP, Gal-3, HSP-27, or NT-proBNP with gestational hypertension and/or preeclampsia. 16](#_Toc146972329)

[eTable 6. Disease traits analyzed in the phenome-wide MR analyses. 16](#_Toc146972330)

[eTable 7. Genetic variants included in the genetic instruments for all proteins with valid *cis*-pQTLs (*n*=75). 17](#_Toc146972331)

[eTable 8. Associations of genetically predicted protein levels with gestational hypertension in primary *cis*-MR analyses. 17](#_Toc146972332)

[eTable 9. Associations of genetically predicted protein levels with preeclampsia in primary *cis*-MR analyses. 17](#_Toc146972333)

[eTable 10. Associations of genetically predicted protein levels with gestational hypertension and preeclampsia in sensitivity analyses. 17](#_Toc146972334)

[eTable 11. Mendelian randomization (MR) analyses testing the associations of gestational hypertension and preeclampsia with circulating levels of CD40, CSTB, ECP, Gal-3, HSP-27, or NT-proBNP (i.e., opposite direction of effects). 18](#_Toc146972335)

[eTable 12. Replication of the associations of genetically predicted protein levels with gestational hypertension and preeclampsia in the UK Biobank Pharma Proteomics Project (UKB-PPP). 18](#_Toc146972336)

[eTable 13. Colocalization of *cis*-protein quantitative trait loci (*cis*-pQTLs) of CD40, CSTB, ECP, Gal-3, HSP-27, and NT-proBNP with gestational hypertension or preeclampsia. 18](#_Toc146972337)

[eTable 14. Characteristics of the included studies reporting observational associations of CD40, CSTB, ECP, Gal-3, HSP-27, or NT-proBNP with gestational hypertension and/or preeclampsia. 19](#_Toc146972338)

[eTable 15. Characteristics of participants included in studies reporting observational associations of CD40, CSTB, ECP, Gal-3, HSP-27, or NT-proBNP with gestational hypertension and/or preeclampsia. 19](#_Toc146972339)

[eTable 16. Observational associations of CD40, CSTB, ECP, Gal-3, HSP-27, and NT-proBNP with gestational hypertension and preeclampsia. 19](#_Toc146972340)

[eTable 17. Full results from phenome-wide Mendelian randomization (MR) analyses evaluating potential on-target side effects associated with therapeutic interventions on the identified proteins. 19](#_Toc146972341)

[eTable 18. Significant results from phenome-wide Mendelian randomization (MR) analyses evaluating potential on-target side effects associated with therapeutic interventions on the identified proteins. 20](#_Toc146972342)

[eTable 19. Drug compounds targeting CD40, CSTB, ECP, Gal-3, HSP-27, NT-proBNP, or GC-A that are currently in development or approved for clinical use. 20](#_Toc146972343)

[References 21](#_Toc146972344)

# eMethods

## Assumptions of MR and the use of *cis*-pQTLs

Causal inference using MR relies on 3 assumptions (**Supplemental Figure 2**).^1^ First, a genetic instrument must be associated with the exposure of interest (relevance assumption). Second, there must be no unmeasured confounders that could affect the associations between the genetic instrument and outcomes (independence assumption). Third, the genetic instrument can only affect the outcome through its effect on the exposure (i.e., exclusion restriction assumption or absence of horizontal pleiotropy). The use of *cis*-pQTLs as genetic instruments facilitates adherence to these assumptions, since variants located near the protein-encoding gene often have large effects on protein expression in comparison to other traits and are less prone to violating the "no horizontal pleiotropy" assumption than variants located elsewhere in the genome.^2,3^ In addition, MR analyses using *cis­*-pQTLs reduce the risk of reverse causation because it is more likely that *cis*-pQTLs influence protein levels, which then influence a disease, rather than the disease influencing protein levels through *cis*-pQTLs.^2,3^

## Measurement and GWAS of plasma protein levels

Genetic variants associated with circulating cardiovascular disease-related protein levels were used as the exposure across all *cis*-MR analyses. Protein levels were measured using the Olink cardiovascular I multiplex proximity extension assay (Olink CVD-I).^4,5^ The Olink CVD-I panel includes 92 protein biomarkers linked to cardiovascular risk or prognosis in human studies and/or animal models, selected by a group of expert cardiovascular disease researchers.^4,5^ The panel was subsequently pruned down based on the availability of high-quality antibodies and relative abundance of the proteins in human plasma. The Olink CVD-I assay uses proximity extension technology, whereby oligonucleotide-labeled antibodies bind to their circulating target proteins. Protein concentrations are then quantified using real-time polymerase chain reaction, and all protein levels are log_2_-transformed by default to reduce technical variation between plates.^4,5^

Genetic association data for the Olink CVD-I proteins were obtained from a meta-analysis by Folkersen *et al.*^5^*,* which included data from up to 13 cohorts and 21,758 individuals of European ancestry from the SCALLOP consortium (Systematic and Combined Analysis of Olink Proteins; <https://www.scallop-consortium.com/>). Plasma protein levels were log_2_-transformed by default and standardized using various study-specific methods, including rank-based standardization, inverse-normal transformation, and standardization to unit variance. Genotyping arrays, imputation strategies, and adjustments specific to individual studies are detailed in **eTable 1**. Proteins that were not detected by the Olink CVD-I assay in ≥20% of samples were excluded from GWAS meta-analysis,^5^ leaving data on 90 of the 92 proteins for analysis (**eTable 2**). Data used from the SCALLOP consortium are publicly available, and ethical approval and informed consent were obtained in each original study.

## GWAS of gestational hypertension and preeclampsia

Genetic variants associated with HDPs were used as the outcome throughout *cis*-MR analyses. Summary statistics were obtained from a GWAS meta-analysis by Honigberg *et al*.^6^ for gestational hypertension (modeled as women with gestational hypertension vs. those without any HDP) and preeclampsia/eclampsia (modeled as women with preeclampsia or eclampsia vs. those without any HDP) separately. Those who had diagnostic codes for both preeclampsia and gestational hypertension were labeled as preeclampsia cases. The present study used the genetic association results for individuals of European ancestry, which included data from 7 studies on up to 393,238 females (8,636 cases and 384,602 controls) for gestational hypertension and from 9 studies on up to 606,903 females (16,032 cases and 590,871 controls) for preeclampsia. Genotyping arrays and imputation schemes varied across the included cohorts (**eTable 3**). Study-specific covariates included age, age^2^, genotype array/batch, and up to 20 principal components of ancestry. Meta-analysis was carried out using fixed-effects inverse-variance weighting. The Estonian Biobank was included in both the GWAS meta-analyses of HDPs and plasma proteins, potentially resulting in overlap of up to 496 individuals (<1% of the meta-analyzed HDP cohort; ~2% of the meta-analyzed biomarker cohort). However, because overlap in a very small proportion of the samples (e.g., <5%) has little effect on the causal estimates, no additional steps were undertaken to adjust for this overlap.^7^ Data used from the GWAS meta-analyses by Honigberg *et al*. are publicly available, and ethical approval and informed consent were obtained in each original study.

## Construction of genetic instruments

To ensure that the positions of variants in the outcome (i.e., HDPs) summary statistics were compatible with those in the exposure (i.e., plasma biomarkers) summary statistics, we used UCSC liftOver (<https://genome.ucsc.edu/cgi-bin/hgLiftOver>) to lift the HDP summary statistics from hg38 to hg19. We used dbSNP (build 151) to assign rsIDs to the variants based on their position-allele combinations and matched the variants between each pair of exposure-outcome summary statistics.

Genetic instruments for plasma proteins were constructed using variants located within or near the gene encoding the protein of interest (i.e., variants *in cis*). Variants were considered to be *in cis* if they mapped within 200 kilobases of the protein-encoding transcription start and stop sites,^8,9^ which were obtained from the Ensembl database (<https://grch37.ensembl.org/>). All variants mapping to the *cis*-region that were also present in the outcome summary statistics were extracted from the protein summary statistics and clumped into independent loci using PLINK 1.9 (<https://www.cog-genomics.org/plink/1.9/>). We lowered the conventional genome-wide significance *P*-value threshold for instrument selection to *P*<1×10^-4^ to increase the number of genetic instruments since a *cis*-region only constitutes a small fraction of the genome.^8^ More conservative *P*-value thresholds (i.e., *P*<1×10^-6^ and *P*<5×10^-8^), which might potentially enhance instrument strength at the expense of missing sub-genome-wide significant pQTLs and yielding fewer proteins with genetic instruments, were tested in sensitivity analyses. In addition, the genetic instruments used in primary analyses were clumped with a more lenient linkage disequilibrium threshold (*R^2^*<0.4; based on simulation studies suggesting that unstable estimates due to multicollinearity started to occur at *R^2^*=0.36)^8,10^ and tested with MR models that account for residual correlation.^8,10,11^ Other linkage disequilibrium *R*² thresholds (*R*²<0.001, *R*²<0.01, *R*²<0.1, *R*²<0.2, *R*²<0.4, *R*²<0.6, and *R*²<0.8) were tested in sensitivity analyses. Primary and sensitivity analyses used a linkage disequilibrium reference panel calculated in 5,000 randomly selected European ancestry samples from the UK Biobank imputed with the Haplotype Reference Consortium panel.^12^

## *Cis-*Mendelian randomization analyses

Consistent with previous *cis*-MR studies,^8,13^ we used different methods to estimate causal effects depending on the number of variants included in each protein's genetic instrument. We used the inverse-variance-weighted (IVW) method with fixed effects for instruments with 2-3 variants and multiplicative random effects for instruments with >3 variants. When the instrument included a single variant, we used the Wald ratio method. In addition, to avoid spurious associations due to residual correlation between variants, we adjusted for between-variant correlation structure in all primary IVW models as described previously.^10,11^ Two-sided *P*<0.05 was used to define statistical significance for the primary analyses, and the Benjamini-Hochberg method was used to correct for multiple comparisons (75 proteins for 2 outcomes) at a false discovery rate (FDR) of 5%.

## Sensitivity analyses

Multiple sensitivity analyses were conducted to probe robustness of our findings using different instrument selection parameters and MR methods.^8^ First, sensitivity analyses using different correlation thresholds are routinely recommended since *cis*-MR analyses often rely on variants that are moderately correlated with each other.^14^ Therefore, we constructed genetic instruments using different correlation thresholds (*R*²<0.001, *R*²<0.01, *R*²<0.1*, R*²<0.2*, R*²<0.4*, R*²<0.6*, R*²<0.8) and tested the associations of these instruments with HDPs. Second, additional sensitivity analyses tested the associations of genetic instruments constructed using stricter *P*-value thresholds (*P*<1×10^-4^, *P*<1×10^-6^, *P*<5×10^-8^) with HDPs. Third, we conducted analyses using MR models with principal components explaining 99% of the genetic variance. The use of principal components in MR ensures that all variants are included in the analysis and weighted by their genetic correlation with other variants.^10,14^ While MR analyses with principal components provide estimates that are more robust than those from models using fixed correlation thresholds (e.g., *R*²<0.4), these are less precise. Fourth, we calculated effect estimates using MR-Egger, a method that also accounts for horizontal pleiotropy. Effect estimates using MR-Egger were adjusted for residual correlation between variants,^11^ consistent with the primary analyses. Finally, we evaluated the possibility of reverse causation affecting our analyses by (1) performing Steiger filtering to remove variants explaining more variance in the outcome than the exposure and (2) testing the genetic associations of HDPs (exposure) with the indicated proteins (outcome). In the latter set of analyses, genetic instruments were constructed using genome-wide significant (*P*<5×10^-8^), strictly uncorrelated (*R*²<0.001) variants associated with preeclampsia or gestational hypertension. Associations of HDPs with the indicated proteins were tested using the conventional IVW method.

Associations of proteins with HDPs were considered robust if: (1) the primary analysis was statistically significant; (2) all sensitivity analyses were directionally consistent; and (3) there was no evidence of reverse causation (unadjusted *P*>0.05).

## Replication analyses in the UK Biobank Pharma Proteomics Project

To further evaluate the robustness of our findings, we performed replication analyses using pQTL data from the UK Biobank Pharma Proteomics Project (UKB-PPP). In brief, the UKB-PPP is a large-scale collaboration between 13 biopharmaceutical companies funding the generation of proteomic data in UK Biobank participants.^15^ A total of 54,306 UK Biobank participants who donated blood samples at baseline and/or follow-up study visits were included in the UKB-PPP cohort. Blood samples were analyzed using proximity extension assay technology (Olink Explore 1536 platform), aligning with the protein assay used in the SCALLOP consortium.

Sun *et al*.^15^ recently performed a GWAS of all proteins measured using this assay in the UKB-PPP, using a discovery sample of 35,571 individuals of European ancestry. All 35,571 participants included in the discovery sample were part of a subset of UKB-PPP participants who were randomly selected from the full UK Biobank cohort (*n*=46,673 of 54,306 UKB-PPP participants [85.9%]); the remaining participants were selected by UKB-PPP consortium members (*n*=6,385 [11.8%]) or included because they participated in the COVID-19 repeat imaging study (*n*=1,268 [2.3%]). Demographics of the "randomized subset" have been published previously^15^ and are detailed in **eTable 4**. Additional details on the UKB-PPP's study design, protein measurements, and pQTL mapping have been described previously.^15^

Consistent with the primary analyses, replication analyses used pQTL data derived from European ancestry participants (*n*=35,571).^15^ Replication analyses used the same outcome (i.e., gestational hypertension and preeclampsia) summary statistics,^6^ *cis*-instrument selection parameters (±200 kilobases, *P*<1×10^-4^, *R*²<0.4), and MR methods (IVW adjusting for between-variant correlation structure) as those used in primary analyses. *P*<0.05 and directional consistency with the primary *cis*-MR analysis indicated replication.

## Colocalization analyses

Colocalization analyses tested for shared causal variants between the prioritized proteins' *cis* loci (obtained from the SCALLOP consortium^5^ and UKB-PPP cohort^15^) and HDPs (obained from Honigberg *et al*.^6^). Analyses were performed with window sizes both at ±0 and ±200 kilobases from each biomarker's protein-encoding region, using the *coloc.abf()* function from the R package *coloc* (version 5.2.2).^16^ The results from these colocalization analyses were expressed as test statistics estimating the posterior probabilities of five hypotheses: *H*_0_, neither trait has an association with a genetic variant in the region; *H*_1_, only the indicated protein measurement has an association with a genetic variant in the region; *H*_2_, only the indicated HDP has an association with a genetic variant in the region; *H*_3_, both traits are associated but with different causal variants; and *H*_4_, both traits are associated and share a single causal variant. A posterior probability for *H*_4_ >0.80 indicated strong evidence of colocalization.

## Systematic review of observational data

Next, we evaluated whether our findings were consistent with observational data. We conducted a comprehensive literature search in PubMed/MEDLINE using free-text and index terms listed in **eTable 5**. A total of 413 potential citations were identified, of which 114 were selected for full-text review (**eFigure 3**). We excluded animal and *in vitro* studies, review articles, letters, editorials, conference abstracts, and studies written in non-English language. Studies were included if they: (1) reported data on at least one of the proteins of interest (i.e., proteins that were robust to sensitivity analyses); (2) reported circulating protein concentrations or fold changes in circulating protein concentrations; (3) compared circulating protein concentrations between pregnant women with vs. without HDPs; and (4) were not restricted to a certain population (e.g. women with twin pregnancies or those with pre-existing cardiovascular disease). Whenever there was potential sample overlap between studies, we only included the study with the largest sample size.

To facilitate comparisons between studies, we used a fold-change approach to quantify alterations in protein abundance in women with vs. those without HDPs. To do so, we log_10_-transformed the ratio of the mean protein concentration in the HDP group to that in the non-HDP group. When possible, we extracted data on HDP subtype (i.e., gestational hypertension or preeclampsia/eclampsia) and reported protein measurements for each subgroup separately. Because previous research suggests that associations of biomarkers with HDPs can change over the course of pregnancy,^17^ we evaluated trends in protein abundance in women with vs. without HDPs over time, when possible. Putative trends were evaluated using linear regression analyses weighted by each study's sample size. Additional analyses visualized these putative trends using loess (i.e., locally weighted) smoothing to interrogate potential nonlinear relationships.

## Phenome-wide MR analyses

Proteins robust to sensitivity analyses were selected for phenome-wide MR analyses to investigate potential non-HDP-related effects (i.e., "on-target" side effects). *Cis*-instruments (±200 kilobases, *P*<1×10^-4^, *R*²<0.4) were tested against genetic association data for 708 phecode-based disease traits, derived from up to 408,961 European-ancestry UK Biobank participants (**eTable 6**).^18^ Phecodes represent groups of diagnostic codes (e.g., *International Classification of Diseases*), grouped by organ system, disease, and/or clinical features.^19^ We excluded sex-specific diseases and diseases with fewer than 500 cases due to lack of statistical power, as done previously.^20^ To frame results in a clinical context, we aligned all genetic instruments with the direction of effects that corresponded to a reduction in HDP risk. For instance, if lower protein levels were associated with a higher risk of HDPs (i.e., *β*<0 in primary analyses), associations were expressed per 1-SD increase in genetically predicted protein levels, and vice versa. Phenome-wide MR analyses were performed using the IVW method adjusting for between-variant correlation structure. To increase the sensitivity of our analyses for detecting potential side effects, we applied a more lenient *P*-value threshold for statistical significance (*P*<0.0083 [i.e., 0.05/6]), correcting solely for the number of proteins tested and not for the number of disease traits. A protein-disease association was considered "adverse" if genetically predicted alterations in protein levels, consistent with reduced HDP risk, were associated with a higher risk of the corresponding disease. An association was considered "beneficial" if similar genetically predicted alterations in protein levels were associated with a lower risk of the corresponding disease.

## Assessment of druggability profiles

Druggability profiles of potential target proteins were extracted from an updated list of druggable genes,^21^ as done previously.^8^ In brief, Finan *et al*.^21^ constructed this list of druggable genes by stratifying 20,300 human protein-encoding genes into 4 categories. The first category (*n*=1,427 genes) included all genes encoding targets of approved drugs or drugs in clinical development, based on information obtained from biochemical databases (e.g., ChEMBL^22,23^), large pharmaceutical companies' investor pipelines, drug name applications, and clinical trial protocols. This first category also incorporated genes involved in drug absorption, distribution, metabolism, and excretion, identified from PharmaADME's extended list ([www.pharmaadme.org](http://www.pharmaadme.org)). The second category (*n*=682) included proteins that were closely related to drug targets (sharing ≥50% identity across ≥75% of their peptide sequence), as well as proteins with associated small-molecule drug-like binding partners (queried using ChEMBL). The third category (*n*=2,370) included members of key drug target families (i.e., G protein-coupled receptors, kinases, ion channels, nuclear hormone receptors, and phosphodiesterases) and extracellular or secreted proteins, identified using a range of biochemical databases.^21^ All genes included in categories 1 through 3 were considered to be druggable (*n*=4,479); the remainder of protein-encoding genes were assigned to a fourth category for non-druggable genes (*n*=15,821). Additional details on this list of druggable genes are provided by Finan *et al*.^21^

Furthermore, we utilized the ChEMBL database (version 32; <https://www.ebi.ac.uk/chembl/>),^22,23^ ClinicalTrials.gov (<https://clinicaltrials.gov/>), and the *Drug Gene Interaction database* (DGIdb; <https://www.dgidb.org/>) to assess the clinical development status of candidate drugs targeting these proteins. For proteins that were associated with higher risk of developing HDPs through lower genetically predicted levels (i.e., *β*<0 in primary analyses), we also examined the availability of any agonist drugs mimicking the effects of the target proteins.

## Statistical software and packages

MR analyses were performed in R (version 4.1.0) using the *TwoSampleMR* and *MendelianRandomization* packages.^24,25^

# eFigures

##
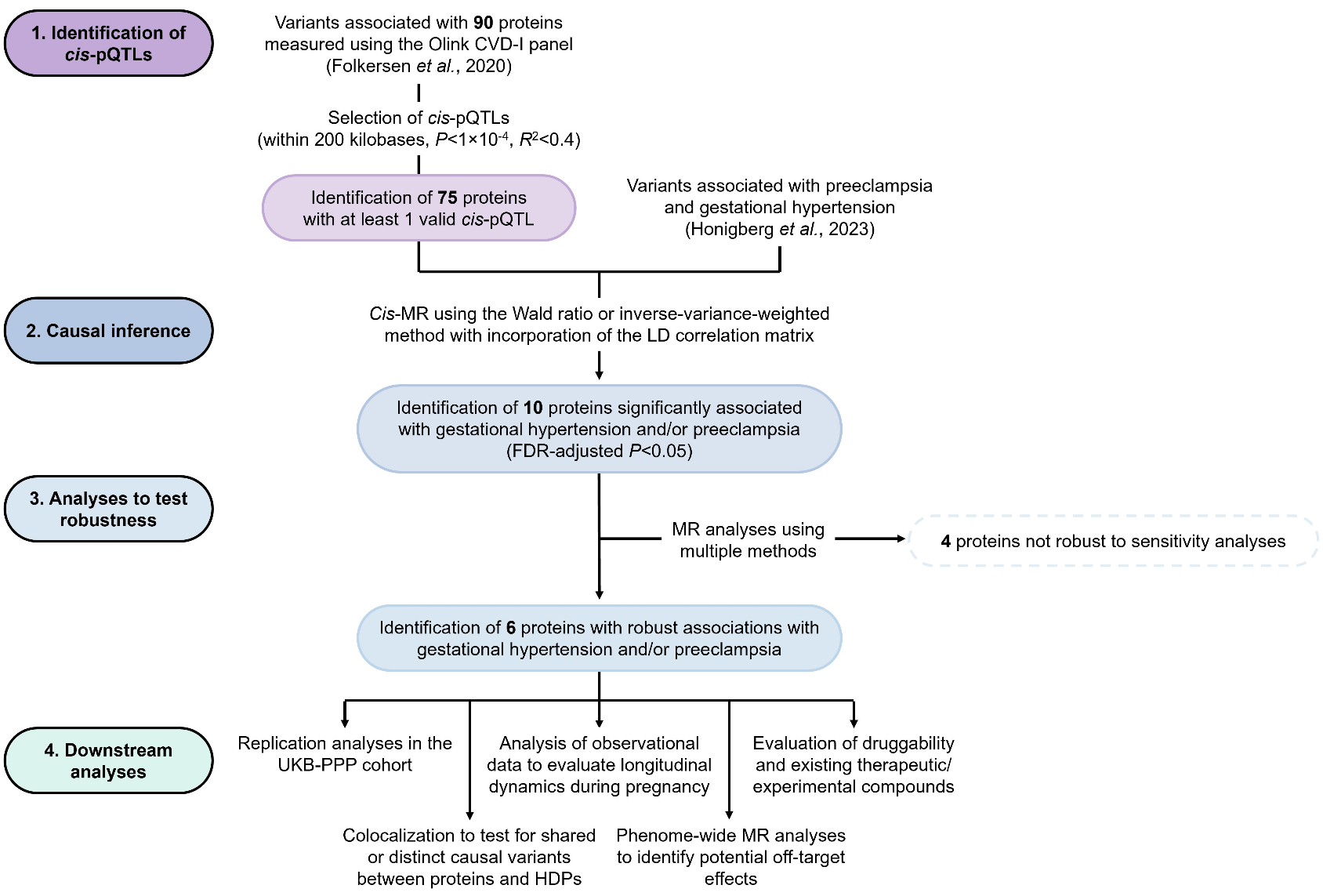
eFigure 1. Study design.

FDR, false discovery rate; HDP, hypertensive disorder of pregnancy; LD, linkage disequilibrium; MR, Mendelian randomization; pQTL, protein quantitative trait locus; UKB-PPP, UK Biobank Pharma Proteomics Project.

##
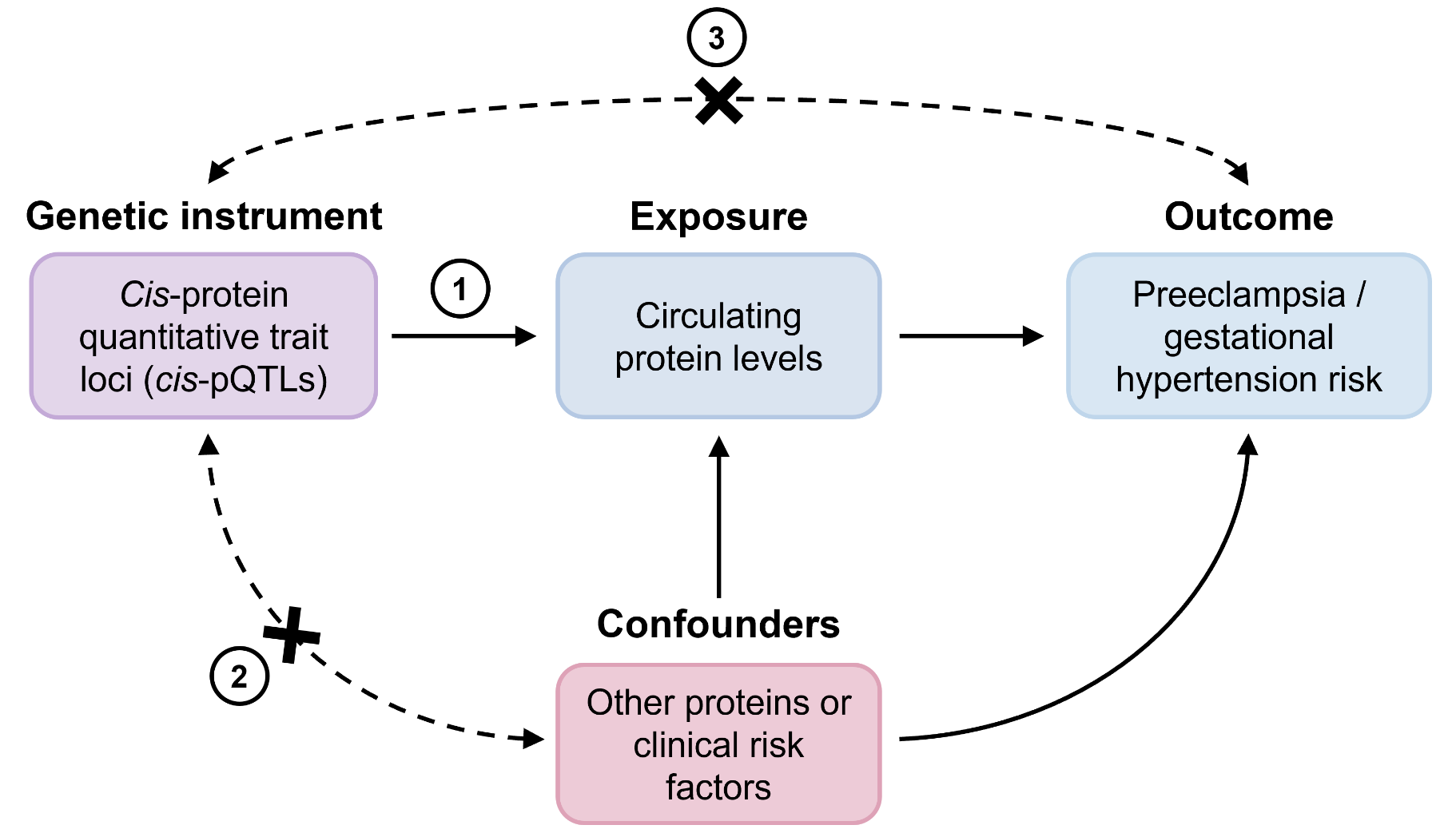
eFigure 2. Schematic illustration of the *cis*-Mendelian randomization (*cis*-MR) framework.

MR relies on three assumptions: (1) genetic instrument must be associated with the exposure of interest; (2) there must be no confounders affecting the associations between the genetic instrument and outcomes; (3) the genetic instrument can only affect the outcome through its effect on the exposure (i.e., "no horizontal pleiotropy"). *Cis*-protein quantitative trait loci (*cis*-pQTLs) facilitate adherence to these assumptions, as further detailed in the **Supplemental Methods**.^8^

##
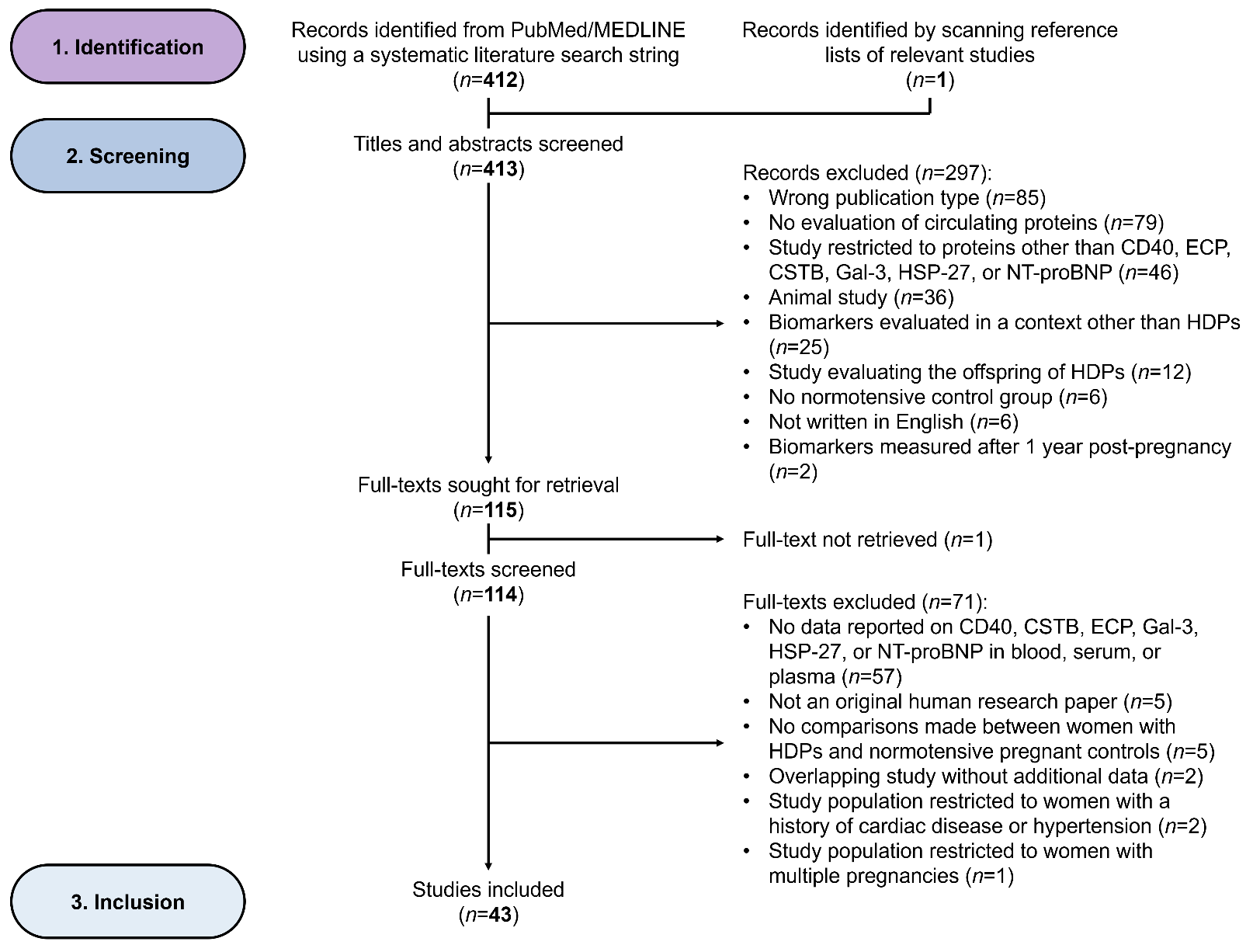
eFigure 3. Flow diagram of the literature search performed to identify studies reporting observational associations of CD40, CSTB, ECP, Gal-3, HSP-27, or NT-proBNP with hypertensive disorders of pregnancy (HDPs).

HDP indicates hypertensive disorder of pregnancy.

##
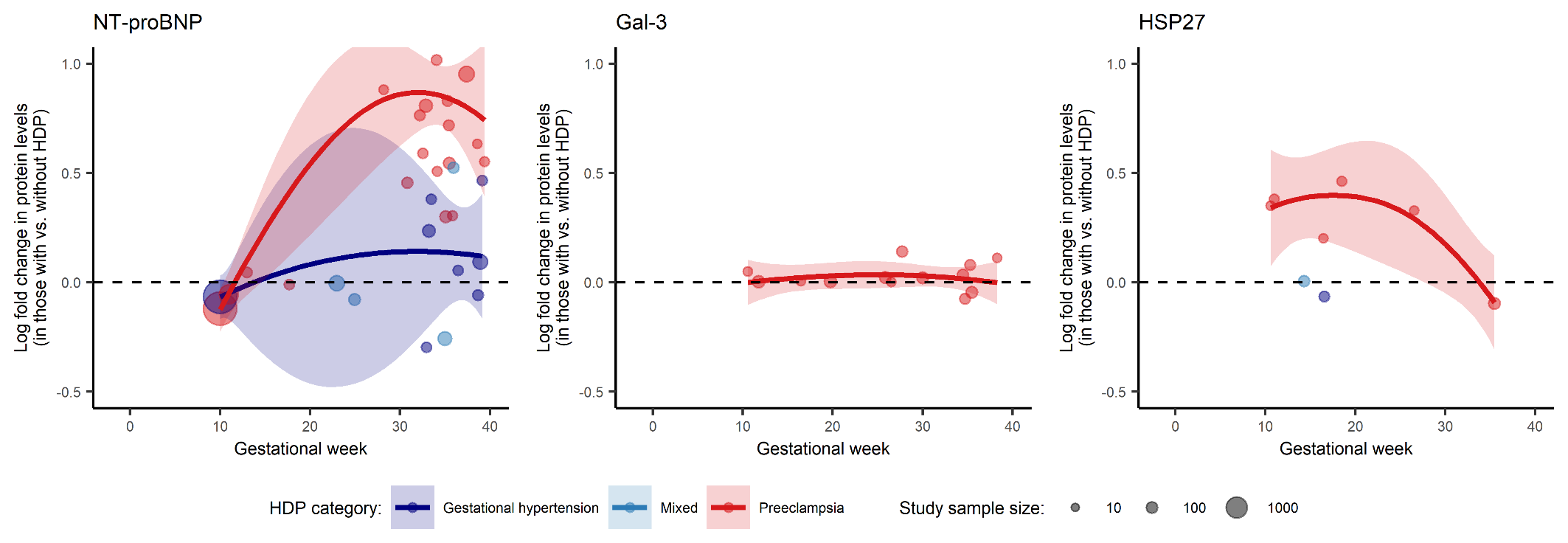
eFigure 4. Observational associations between HDPs and NT-proBNP, Gal-3, and HSP27 across gestation, modeled using loess-smoothed averages.

Scatter plots illustrate the relationship between protein levels and gestational age at blood sampling. Protein levels were compared by log_10_-transforming the ratio of mean protein concentration in the HDP vs. non-HDP group. Curves depict loess-smoothed averages (and corresponding 95% confidence bands) weighted by each study's sample size, generated using *ggplot2* in R.

# eTables (legends)

All eTables can be found in the supplementary Excel file.

## eTable 1. Description of cohorts included in the GWAS meta-analysis of circulating protein levels from the SCALLOP consortium.

This table is adapted from Folkersen *et al.*^5^ GWAS indicates genome-wide association study; IBD, identical by descent; PMID, PubMed ID.

## eTable 2. Description of Olink CVD-I proteins included in the present study.

Start and stop positions were obtained from Ensembl, using *Genome Reference Consortium Human Build 37* (GRCh37; <https://grch37.ensembl.org/>).

## eTable 3. Description of cohorts included in the GWAS meta-analysis of gestational hypertension and preeclampsia/eclampsia.

This table is adapted from Honigberg *et al*.^6^ ICD indicates *International Classification of Diseases*; PMID, PubMed ID. ^a^Preeclampsia/eclampsia: 938005, 41114007, 46764007, 95605009, 161806007, 161807003, 198983002, 198984008, 198985009, 198986005, 198990007, 198991006, 198992004, 198993009, 198993009, 237281009, 237282002, 237283007, 303063000, 398254007, 726513006, 736992003, 736993008, 765182005, 105651000119100, 698638005. ^b^Controls: 7888004, 8333008, 9343003, 15467003, 17333005, 21127004, 21243004, 23667007, 48782003, 52483005, 72892002, 169961004, 177184002, 267015005, 275568006. ^c^Gestational hypertension: 48194001, 709881001, 40521000119100.

## eTable 4. Characteristics of UK Biobank Pharma Proteomics Project (UKB-PPP) participants who provided genetic data for replication analyses.

Continuous characteristics are presented as mean ± standard deviation, whereas categorical variables are presented as proportion. This table was adapted from Sun *et al*.^15^

## eTable 5. Systematic search strategy used to identify studies reporting observational associations of CD40, CSTB, ECP, Gal-3, HSP-27, or NT-proBNP with gestational hypertension and/or preeclampsia.

## eTable 6. Disease traits analyzed in the phenome-wide MR analyses.

This table is adapted from Zhou *et al*.^18^ NEC indicates not elsewhere classifiable; NOS, not otherwise specified.

## eTable 7. Genetic variants included in the genetic instruments for all proteins with valid *cis*-pQTLs (*n*=75).

All positions are expressed using *Genome Reference Consortium Human Build 37* (GRCh37; hg19). *F*-statistics were estimated as *β*²/(standard error)². HDP indicates hypertensive disorder of pregnancy; pQTL, protein quantitative trait locus.

## eTable 8. Associations of genetically predicted protein levels with gestational hypertension in primary *cis*-MR analyses.

All analyses represent *cis*-Mendelian randomization (MR) analyses performed using the inverse-variance-weighted (IVW) method (for instruments with two or more variants) or the Wald ratio method (for instruments with a single variant). Genetic instruments were constructed using cis-variants associated with circulating protein levels at *P*<1×10^-4^ that were clumped at *R*²<0.4. Two-sided *P*<0.05 was used to define statistical significance for the primary analyses, and the Benjamini-Hochberg method was used to correct for multiple comparisons with a false discovery rate (FDR) of 5%. All associations are expressed per standard deviation increase in genetically predicted protein levels. CI indicates confidence interval; OR, odds ratio.

## eTable 9. Associations of genetically predicted protein levels with preeclampsia in primary *cis*-MR analyses.

All analyses represent cis-Mendelian randomization (MR) analyses performed using the inverse-variance-weighted (IVW) method (for instruments with two or more variants) or the Wald ratio method (for instruments with a single variant). Genetic instruments were constructed using *cis*-variants associated with circulating protein levels at *P*<1×10^-4^ that were clumped at *R*²<0.4. Two-sided *P*<0.05 was used to define statistical significance for the primary analyses, and the Benjamini-Hochberg method was used to correct for multiple comparisons with a false discovery rate (FDR) of 5%. All associations are expressed per standard deviation increase in genetically predicted protein levels. CI indicates confidence interval; OR, odds ratio.

## eTable 10. Associations of genetically predicted protein levels with gestational hypertension and preeclampsia in sensitivity analyses.

All analyses represent *cis*-Mendelian randomization (MR) analyses performed using the indicated methods. Genetic instruments were constructed using *cis*-variants associated with circulating protein levels at the indicated *P*-value and *R*² thresholds. Analyses using the inverse-variance-weighted (IVW) method incorporating principal components used genetic instruments that were not clumped against a correlation threshold. Associations were considered robust if the primary analysis was statistically significant (false discovery rate [FDR]-adjusted *P*<0.05) and all sensitivity analyses were directionally consistent. N/A indicates that the variance estimate (which is required to calculate MR estimates that account for between-variant correlation structure) was negative, precluding accurate estimation of confidence intervals. CI indicates confidence interval; HDP, hypertensive disorder of pregnancy; LD, linkage disequilibrium; OR, odds ratio.

## eTable 11. Mendelian randomization (MR) analyses testing the associations of gestational hypertension and preeclampsia with circulating levels of CD40, CSTB, ECP, Gal-3, HSP-27, or NT-proBNP (i.e., opposite direction of effects).

All analyses represent conventional MR analyses using the inverse-variance-weighted method. Genetic instruments were constructed variants associated with HDPs at *P*<5×10^-8^ that were clumped at *R*²<0.001. CI indicates confidence interval; HDP, hypertensive disorder of pregnancy.

## eTable 12. Replication of the associations of genetically predicted protein levels with gestational hypertension and preeclampsia in the UK Biobank Pharma Proteomics Project (UKB-PPP).

All analyses represent *cis*-Mendelian randomization (MR) analyses performed using the inverse-variance-weighted (IVW) method adjusting for between-variant correlation structure. Genetic instruments were constructed using *cis*-variants associated with circulating protein levels at *P*<1×10^-4^ that were clumped at *R*²<0.4. Two-sided *P*<0.05 was used to define replication. All associations are expressed per standard deviation increase in genetically predicted protein levels. CI indicates confidence interval; OR, odds ratio.

## eTable 13. Colocalization of *cis*-protein quantitative trait loci (*cis*-pQTLs) of CD40, CSTB, ECP, Gal-3, HSP-27, and NT-proBNP with gestational hypertension or preeclampsia.

Colocalization analyses were performed for all protein-disease associations that were robust to sensitivity analyses, using *cis*-variants (i.e., using window sizes of ±0 and ±200 kilobases) associated with circulating protein levels obtained from the SCALLOP consortium and UKB-PPP cohort. The current table shows posterior probabilities for *H*_0_ (neither trait has a genetic association in the region), *H*_1_ (only trait 1 [i.e., the indicated protein's circulating levels] has a genetic association in the region), *H*_2_ (only trait 2 [i.e., the indicated hypertensive disorder of pregnancy] has a genetic association in the region), *H*_3_ (both traits are associated but with different causal variants), and *H*_4_ (both traits are associated and share a single causal variant).

## eTable 14. Characteristics of the included studies reporting observational associations of CD40, CSTB, ECP, Gal-3, HSP-27, or NT-proBNP with gestational hypertension and/or preeclampsia.

DOI indicates digital object identifier; HDP, hypertensive disorder of pregnancy.

## eTable 15. Characteristics of participants included in studies reporting observational associations of CD40, CSTB, ECP, Gal-3, HSP-27, or NT-proBNP with gestational hypertension and/or preeclampsia.

If studies reported data on multiple blood sampling time points or stratified by HDP subgroup, we reported these data in separate rows. Author names in bold indicate the first occurrence of that study. All data are summarized as mean ± standard deviation. For studies that did not report mean values, we reported the corresponding median value (if available). When no standard deviations were reported, we estimated standard deviation by dividing the corresponding quartile range by 1.35 (if available). ^a^The exact number of participants included by Freitag *et al.* (2019) is not available. The number of individuals included in the HDP and control groups are both in the range of *n*=9-14.

## eTable 16. Observational associations of CD40, CSTB, ECP, Gal-3, HSP-27, and NT-proBNP with gestational hypertension and preeclampsia.

If studies reported data on multiple blood sampling time points or stratified by HDP subgroup, we reported these data in separate rows. Author names in bold indicate the first occurrence of that study. All data are summarized as mean ± standard deviation. For studies that did not report mean values, we reported the corresponding median value (if available). When no standard deviations were reported, we estimated standard deviation by dividing the corresponding quartile range by 1.35 (if available). HDP indicates hypertensive disorder of pregnancy. ^a^Logarithm with base 10. ^b^The exact number of participants included by Freitag *et al.* (2019) is not available. The number of individuals included in the HDP and control groups are both in the range of *n*=9-14.

## eTable 17. Full results from phenome-wide Mendelian randomization (MR) analyses evaluating potential on-target side effects associated with therapeutic interventions on the identified proteins.

N/A indicates that the variance estimate (which is required to calculate MR estimates that account for between-variant correlation structure) was negative, precluding accurate estimation of confidence intervals.

## eTable 18. Significant results from phenome-wide Mendelian randomization (MR) analyses evaluating potential on-target side effects associated with therapeutic interventions on the identified proteins.

## eTable 19. Drug compounds targeting CD40, CSTB, ECP, Gal-3, HSP-27, NT-proBNP, or GC-A that are currently in development or approved for clinical use.

^a^GC-A (encoded by *NPR1*) represents a drug target that is targeted by compounds mimicking brain natriuretic peptide (BNP).

# References

1. Davies NM, Holmes M V., Davey Smith G. Reading Mendelian randomisation studies: a guide, glossary, and checklist for clinicians. *BMJ*. 2018;362:601. doi:10.1136/BMJ.K601

2. Swerdlow DI, Kuchenbaecker KB, Shah S, Sofat R, Holmes M V., White J, Mindell JS, Kivimaki M, Brunner EJ, Whittaker JC, et al. Selecting instruments for Mendelian randomization in the wake of genome-wide association studies. *Int J Epidemiol*. 2016;45:1600-1616. doi:10.1093/IJE/DYW088

3. Schmidt AF, Finan C, Gordillo-Marañón M, Asselbergs FW, Freitag DF, Patel RS, Tyl B, Chopade S, Faraway R, Zwierzyna M, et al. Genetic drug target validation using Mendelian randomisation. *Nat Commun*. 2020;11:1-12. doi:10.1038/s41467-020-16969-0

4. Assarsson E, Lundberg M, Holmquist G, Björkesten J, Thorsen SB, Ekman D, Eriksson A, Dickens ER, Ohlsson S, Edfeldt G, et al. Homogenous 96-Plex PEA Immunoassay Exhibiting High Sensitivity, Specificity, and Excellent Scalability. *PLoS One*. 2014;9:e95192. doi:10.1371/JOURNAL.PONE.0095192

5. Folkersen L, Gustafsson S, Wang Q, Hansen DH, Hedman ÅK, Schork A, Page K, Zhernakova D V., Wu Y, Peters J, et al. Genomic and drug target evaluation of 90 cardiovascular proteins in 30,931 individuals. *Nat Metab*. 2020;2:1135-1148. doi:10.1038/s42255-020-00287-2

6. Honigberg MC, Truong B, Khan RR, Xiao B, Bhatta L, Vy HMT, Guerrero RF, Schuermans A, Selvaraj MS, Patel AP, et al. Polygenic prediction of preeclampsia and gestational hypertension. *Nat Med*. Published online May 29, 2023:1-10. doi:10.1038/s41591-023-02374-9

7. Burgess S, Davies NM, Thompson SG. Bias due to participant overlap in two-sample Mendelian randomization. *Genet Epidemiol*. 2016;40:597-608. doi:10.1002/GEPI.21998

8. Henry A, Gordillo-Marañón M, Finan C, Schmidt AF, Ferreira JP, Karra R, Sundström J, Lind L, Arnlöv J, Zannad F, et al. Therapeutic Targets for Heart Failure Identified Using Proteomics and Mendelian Randomization. *Circulation*. 2022;145:1205-1217. doi:10.1161/CIRCULATIONAHA.121.056663

9. Sun BB, Maranville JC, Peters JE, Stacey D, Staley JR, Blackshaw J, Burgess S, Jiang T, Paige E, Surendran P, et al. Genomic atlas of the human plasma proteome. *Nature*. 2018;558:73-79. doi:10.1038/s41586-018-0175-2

10. Burgess S, Zuber V, Valdes-Marquez E, Sun BB, Hopewell JC. Mendelian randomization with fine‐mapped genetic data: Choosing from large numbers of correlated instrumental variables. *Genet Epidemiol*. 2017;41:714. doi:10.1002/GEPI.22077

11. Burgess S, Dudbridge F, Thompson SG. Combining information on multiple instrumental variables in Mendelian randomization: comparison of allele score and summarized data methods. *Stat Med*. 2016;35:1880-1906. doi:10.1002/SIM.6835

12. Bycroft C, Freeman C, Petkova D, Band G, Elliott LT, Sharp K, Motyer A, Vukcevic D, Delaneau O, O’Connell J, et al. The UK Biobank resource with deep phenotyping and genomic data. *Nature*. 2018;562:203-209. doi:10.1038/s41586-018-0579-z

13. Chen L, Peters JE, Prins B, Persyn E, Traylor M, Surendran P, Karthikeyan S, Yonova-Doing E, Di Angelantonio E, Roberts DJ, et al. Systematic Mendelian randomization using the human plasma proteome to discover potential therapeutic targets for stroke. *Nat Commun*. 2022;13:1-14. doi:10.1038/s41467-022-33675-1

14. Gkatzionis A, Burgess S, Newcombe PJ. Statistical methods for cis-Mendelian randomization with two-sample summary-level data. *Genet Epidemiol*. 2023;47:3-25. doi:10.1002/GEPI.22506

15. Sun BB, Chiou J, Traylor M, Benner C, Hsu YH, Richardson TG, Surendran P, Mahajan A, Robins C, Vasquez-Grinnell SG, et al. Genetic regulation of the human plasma proteome in 54,306 UK Biobank participants. bioRxiv. Published June 18, 2022. Accessed September 6, 2023. https://www.biorxiv.org/content/10.1101/2022.06.17.496443v1

16. Giambartolomei C, Vukcevic D, Schadt EE, Franke L, Hingorani AD, Wallace C, Plagnol V. Bayesian Test for Colocalisation between Pairs of Genetic Association Studies Using Summary Statistics. *PLoS Genet*. 2014;10:e1004383. doi:10.1371/JOURNAL.PGEN.1004383

17. Parry S, Carper BA, Grobman WA, Wapner RJ, Chung JH, Haas DM, Mercer B, Silver RM, Simhan HN, Saade GR, et al. Placental protein levels in maternal serum are associated with adverse pregnancy outcomes in nulliparous patients. *Am J Obstet Gynecol*. 2022;227:497.e1-497.e13. doi:10.1016/J.AJOG.2022.03.064

18. Zhou W, Nielsen JB, Fritsche LG, Dey R, Gabrielsen ME, Wolford BN, LeFaive J, VandeHaar P, Gagliano SA, Gifford A, et al. Efficiently controlling for case-control imbalance and sample relatedness in large-scale genetic association studies. *Nat Genet*. 2018;50:1335-1341. doi:10.1038/s41588-018-0184-y

19. Wu P, Gifford A, Meng X, Li X, Campbell H, Varley T, Zhao J, Carroll R, Bastarache L, Denny JC, et al. Mapping ICD-10 and ICD-10-CM Codes to Phecodes: Workflow Development and Initial Evaluation. *JMIR Med Inform*. 2019;7:e14325. doi:10.2196/14325

20. Chong M, Sjaarda J, Pigeyre M, Mohammadi-Shemirani P, Lali R, Shoamanesh A, Gerstein HC, Paré G. Novel Drug Targets for Ischemic Stroke Identified Through Mendelian Randomization Analysis of the Blood Proteome. *Circulation*. 2019;140:819-830. doi:10.1161/CIRCULATIONAHA.119.040180

21. Finan C, Gaulton A, Kruger FA, Lumbers RT, Shah T, Engmann J, Galver L, Kelley R, Karlsson A, Santos R, et al. The druggable genome and support for target identification and validation in drug development. *Sci Transl Med*. 2017;9. doi:10.1126/SCITRANSLMED.AAG1166/SUPPL_FILE/AAG1166_TABLE_S1.ZIP

22. Mendez D, Gaulton A, Bento AP, Chambers J, De Veij M, Félix E, Magariños MP, Mosquera JF, Mutowo P, Nowotka M, et al. ChEMBL: towards direct deposition of bioassay data. *Nucleic Acids Res*. 2019;47:D930-D940. doi:10.1093/NAR/GKY1075

23. Davies M, Nowotka M, Papadatos G, Dedman N, Gaulton A, Atkinson F, Bellis L, Overington JP. ChEMBL web services: streamlining access to drug discovery data and utilities. *Nucleic Acids Res*. 2015;43:W612-W620. doi:10.1093/NAR/GKV352

24. Hemani G, Zheng J, Elsworth B, Wade KH, Haberland V, Baird D, Laurin C, Burgess S, Bowden J, Langdon R, et al. The MR-base platform supports systematic causal inference across the human phenome. *Elife*. 2018;7. doi:10.7554/ELIFE.34408

25. Yavorska OO, Burgess S. MendelianRandomization: an R package for performing Mendelian randomization analyses using summarized data. *Int J Epidemiol*. 2017;46:1734-1739. doi:10.1093/IJE/DYX034
